# Supplementary material for: Identification of Freezing-Responsive microRNAs and Their Targets in Chinese Jujube by Small RNA and Degradome Sequencing
Source: Int J Mol Sci. 2026 Sep 4;27(17):7912. doi: 10.3390/ijms27177912 (PMC13566890; doi:10.3390/ijms27177912)
Supplement: Supplementary file 1 [file ijms-27-07912-s001.zip › Supplementary Material S2/novel_miR_105.pdf]

Provisional ID	:	novel_miR_105
Score total	:	694
Score for star read(s)	:	3.9
Score for read counts	:	687.9
Score for mfe	:	0.6
Score for randfold	:	1.6
Score for cons. seed	:	
Total read count	:	1361
Mature read count	:	1030
Loop read count	:	2
Star read count	:	329

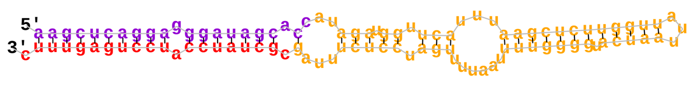

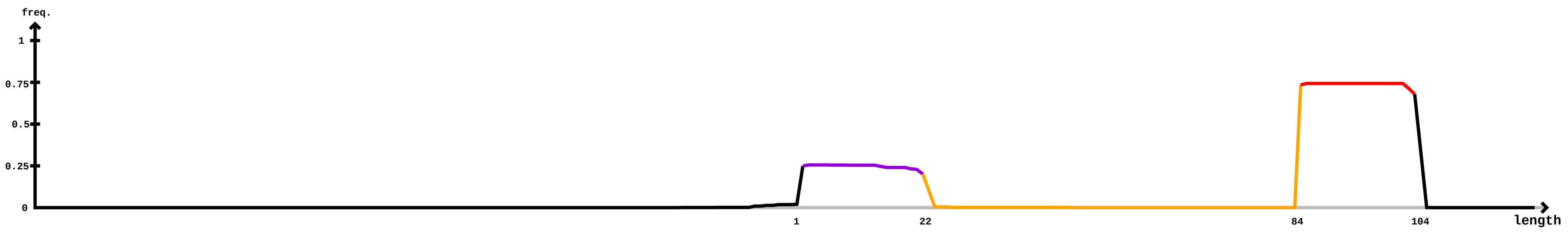

Star Mature

```
5'- ccuuuucucuuaaaagggcucaagcuuuuagccuuugcuuuaaucccuuugcucauaaaacuucauacaaagaaaagagaagaaaauuaaaaagaaaagaaaagaaaaguguuuggagaaucuguaaagcucaggagggauagcaccauagaugguucauuuaagcucuugguuauuaaucaugggguuuuaauuuugauccucuuuagcgcuauccauccugaguuucauagcuucuucuugcucauau -3' obs
    ccuuuucucuuaaaagggcucaagcuuuuagccuuugcuuuaaucccuuugcucauaaaacuucauacaaagaaaagagaagaaaauuaaaaagaaaagaaaagaaaaguguuuggagaaucuguaaagcucaggagggauagcaccauagaugguucauuuaagcucuugguuauuaaucaugggguuuuaauuuugauccucuuuagcgcuauccauccugaguuucauagcuucuucuugcucauau exp
    ..(((((((((.((((((...((((...........))))....)))))).........(((......)))..))))))))).........................(.((..((((((.((((.((((((((((.(((((((.(...(((.((.(((...((((((((((((...))))).)))))))......))).)))))...).)))))))).))))))))))).)))).))))))..)).)....
```

read	reads	mm	sample
aaguguuuggagaaucuguaaagcucag	1	0	LT-3
uuggagaaucuguaaagcu	1	0	LT-1
aaucuguaaagcucaggaggg	8	0	JCK-2
aaucuguaaagcucaggaggga	3	0	LT-1
ucuguaaagcucaggaggg	2	0	JCK-3
ucuguaaagcucaggagggauagc	1	0	LT-1
ucuguaaagcucaggagggauagc	1	0	JCK-1
ucuguaaagcucaggagggauagc	2	0	JCK-3
uguaaagcucaggaggga	6	0	JT-3
Caagcucaggagggauagcac	1	1	JCK-3
aaagcucaggagggauagcacU	1	1	LT-3
aaagcucaggagggauagcacc	1	0	LT-3
aagcucaggagggauagc	2	0	LT-2
aagcucaggagggauagc	3	0	LT-1
aagcucaggagggauagc	1	0	LCK-3
aagcucaggagggauagc	1	0	LT-3
aagcucaggagggauagca	3	0	LT-3
aagcucaggagggauagAa	1	1	LT-2
aagcucaggagggauagcaU	3	1	LT-2
aagcucaggagggauagcac	6	0	LT-3
aagcucaggagggauagcaA	1	1	LT-1
aagcucaggagggauagcac	3	0	LT-1
aagcucaggagggauagcac	5	0	JCK-3
aagcucaggagggauagcac	3	0	LT-2
aagcucaAgagggauagcac	1	1	LT-3
aagcucaggagggauagcac	8	0	LCK-3
aagcucaggagggauagcac	4	0	JCK-1
aagcucaggagggauagcac	4	0	JCK-2
aagcucCggagggauagcacc	1	1	LCK-3
aCgcucaggagggauagcacc	1	1	LT-2
aagcucaggagggauagcacc	13	0	LT-2
aagcucaggagggauagcacc	15	0	LCK-3
aagcucaggagggauagcacU	1	1	LT-1

Star   Mature

ccuuuucucuuaaaagggcucaagcuuuuagccuuugcuuuaaucccuuugcucauaaaacuucauacaaagaaaagagaagaaaauuaaaaagaaaagaaaagaaaaguguuuggagaaucuguaaagcucaggagggauagcaccauagaugguucauuuaagcucuugguuauuaaucaugggguuuuaauuuugauccucuuuagcgcuauccauccugaguuucauagcuucuucuugcucauau			
aagcucaggagggauagcacc	50	0	LT-3
aCgcucaggagggauagcacc	1	1	LCK-3
aagcucaggagggauagcacc	23	0	LT-1
aagcucaAgagggauagcacc	1	1	JCK-2
aagcucaggagggauagcacc	47	0	JCK-3
aagcucaggagggauagcacc	71	0	JCK-2
aagcucaggagggauagcacc	31	0	JCK-1
aagcucaggagggauagcGcca	1	1	LCK-3
aagcucaggagggauagcaccU	3	1	LT-2
aagcucaggagggauagcaccU	6	1	LCK-3
aagcucaggagggauagcaccauUg	2	1	JCK-1
aagcucaggagggauagcaccauUg	2	1	JCK-2
agcucaggagggauagca	1	0	LT-3
agcNcaggagggauagcacc	1	1	JCK-1
agcucaggagggauagcacc	3	0	JCK-1
agcucaggagggauagcacc	1	0	JCK-3
agcucaggagggauagcacc	1	0	JCK-2
agcucaggagggauagcGcca	1	1	LT-3
auagaugguucauuuaagcucuug	2	0	LT-2
cgcuauccauccugaguu	12	0	LT-2
cgcuauccauccugaguu	2	0	JCK-3
cgcuauccauccugaguu	2	0	JCK-1
cgcuauccauccugaguu	19	0	LT-3
cgcuauccauccugaguu	5	0	LT-1
cgcuauccauccugaguu	1	0	JCK-2
cgcuauccauccugaguuu	1	0	LT-1
cgcuauccauccugaguuu	5	0	LT-2
cgcuauccauccugaguuu	6	0	JCK-2
cgcuauccauccugaguuu	8	0	LCK-3
cgcuauccauccugaguuu	10	0	LT-3
cgcuauccauccugaguuA	1	1	JCK-2
cgcuauccauccugaguuu	3	0	JCK-3
cgcuauccauccugaguuu	14	0	JT-1
cgcuauccauccugaguuuc	112	0	JCK-1
cgcuauccauccugaguuuc	7	0	JT-1
cgcuauccauccugaguuuU	8	1	LT-1
cgcuauccauccugaguuuc	161	0	JCK-3
cCcuauccauccugaguuuc	1	1	LT-2
cgcuCuccauccugaguuuc	1	1	LT-2
cgcuauccauccugaguuuU	9	1	JCK-3
cgcuauccauccugaguuuU	6	1	LCK-3
cgcuauccauccugaguuuc	23	0	LCK-3
cgcuauccauccugCguuuc	1	1	JCK-2
cgcuauccauccugaguuGc	1	1	LT-1
cgcuCuccauccugaguuuc	1	1	JCK-1
cgcuauccauccugaguuuc	89	0	LT-3
cgcuauccauccugaguuuU	7	1	LT-2
cgcuauccauccugaguuuA	2	1	LT-2
cgcuauccauccugaguuuc	192	0	JCK-2
cgcuauccauccugaguuuc	45	0	LT-1
cgcuauccauccugaguuuA	1	1	LT-3
cgcuauccauccugaguuuU	5	1	JCK-1
cgcuauccauccugaguuuU	18	1	JCK-2
cAcuauccauccugaguuuc	1	1	LT-2
cgcuauccauccugaguuuc	61	0	LT-2
cgcuaucUauccugaguuuc	1	1	LT-3
cgcuauccauccugaguuuU	14	1	LT-3
cgcuauccauccugaguuuca	4	0	JCK-1
cgcuauccauccugaguuuca	63	0	LCK-2
cgcuauccauccugaguuuca	46	0	LCK-1
cgcuauccauccugaguuucN	1	1	JCK-1
cgcuauccauccugaguuuca	5	0	LT-1
cgcuauccauccugaguuuca	1	0	LT-2
cgcuauccauccugaguuuca	8	0	JCK-3
cgcuauccauccNgaguuuca	1	1	LCK-1
cgcuauccauccugaguuuca	12	0	LCK-3
cgcuauccauccugaguuucG	1	1	LT-3
cgcuauccauccugaguuuca	8	0	LT-3
cgcuauccauccugaguuuca	12	0	JCK-2
cgcuauccauccugaguuucUu	1	1	LT-3

```
                                                                                                                                                  Star                                                                                        Mature
ccuuuucucuuaaaagggcucaagcuuuuagccuuugcuuuaaucccuuugcucauaaaacuucauacaaagaaaagagaagaaaauuaaaaagaaaagaaaagaaaaguguuuggagaaucuguaaagcucaggagggauagcaccauagaugguucauuuaagcucuugguuauuaaucaugggguuuuaauuuugauccucuuuagcgcuauccauccugaguuucauagcuucuucuugcucauau
..................................................................................................................................................................................................................gcuauccauccugaguuuc.....................   6   0   JCK-2
..................................................................................................................................................................................................................gcuauccauccugaguuuc.....................   4   0   JCK-3
..................................................................................................................................................................................................................gcuauccauccugaguuuU.....................   1   1   LT-1
```
